# Circulating Tumor Cells: Who is the Killer?

**DOI:** 10.1007/s12307-014-0164-4

**Published:** 2014-12-20

**Authors:** Patrizia Paterlini-Bréchot

**Affiliations:** 1 Université Paris Descartes, Pavillon Leriche, 75014 Paris, France; 2 INSERM Unit U1151 Eq13, 14, rue Maria Helena Vieira Da Silva, Pavillion Leriche - Porte 14, 75014 Paris, France; 3 Laboratoire de Biochimie, Hôpital Necker Enfants Malades, Paris, France

**Keywords:** Circulating tumor cells, Circulating cancer cells, Circulating epithelial cells, ISET, Cell Search, CTC tests, Tumor invasion

## Abstract

This article is a critical note on the subject of Circulating Tumor Cells (CTC). It takes into account the tumor identity of Circulating Tumor Cells as cancer seeds in transit from primary to secondary soils, rather than as a “biomarker”, and considers the help this field could bring to cancer patients. It is not meant to duplicate information already available in a large number of reviews, but to stimulate considerations, further studies and development helping the clinical use of tumor cells isolated from blood as a modern personalized, non-invasive, predictive test to improve cancer patients’ life. The analysis of CTC challenges, methodological bias and critical issues points out to the need of referring to tumor cells extracted from blood without any bias and identified by cytopathological diagnosis as Circulating Cancer Cells (CCC). Finally, this article highlights recent developments and identifies burning questions which should be addressed to improve our understanding of the domain of CCC and their potential to change the clinical practice.

## Introduction

Some words bear a deadly meaning. The word “killer” means «who can kill ». Likewise, the word “tumor cell” means “potentially tumorigenic”. Killers identification is clearly easier when they are found on the crime scene, alias the primary or metastatic tumor, than if they are in the street. These “killers in the street” are circulating tumor cells (CTC).

Our work and interest in the domain of CTC and the study of the “CTC literature” has generated the view that the possibility to improve cancer patients’ life using CTC- based tests is linked to their unbiased isolation and identification without mistake.

This article is a critical note on CTC which takes into account the tumor identity of Circulating Tumor Cells as cancer seeds in transit from the primary to the secondary soils, and the change that this field could bring to the clinical practice. It is not meant to duplicate information already available in a large number of reviews [1–11], but to stimulate consideration, studies and further development helping the successful clinical use of CTC, as a modern personalized, non-invasive, predictive test to improve cancer patients’ life. Since we focus on a non-invasive test, Disseminated Tumor Cells (DTC), which are tumor cells located in the bone marrow, lymph nodes or distant organs, and angiotropic tumor cells [12], which are tumor cells migrating along the vessels, despite their relevance for the appraisal of tumor invasion, are not in the scope of this article.

## CTC Potential for Clinical Benefit and Key Issue

The spontaneous circulation in blood of tumor cells and/or of tumor microemboli is known to be the hallmark of the tumor’s “invasive character” [13].

Tumor invasion and formation of distant metastasis is known to occur via three major routes: i) blood, ii) lymphatic vessels, and iii) transcoelomic spread into the pleural, pericardial, and abdominal cavities [14], to which Lugassy and Barnhill [12] have added the angiotropic tumor cells invasion. Of these fourth routes, only blood can be exploited to develop an early and non-invasive detection of tumor invasion, which could be of clinical help by taking advantage from the slow and inefficient process leading to metastasis formation [13, 15].

For patients with solid cancer, the sensitive and reliable detection and enumeration of cancer cells in blood is expected to provide a powerful diagnostic tool for early detection of tumor invasion and early assessment of treatment efficacy. Furthermore, genetic tests targeted to circulating cancer cells collected without bias and diagnosed without mistake could allow the kinetic assessment of theranostic/escape DNA mutations in the circulating cancer cell compartment (non-invasive theranostic use of CTC). For subjects at increased risk of developing solid cancers, the ultrasensitive and diagnostic detection of cancer cells in blood could provide a tool for early diagnosis of invasive cancers before they become detectable by imaging. This would be a remarkable step forward to decrease mortality related to invasive cancers, and would require not only diagnostic identification of cancer cells in blood but also characterization analyses to identify the organ from which the cancer cells have spread.

However, the CTC field is far from these goals. Its critical analysis shows that, despite more than 15,190 publications and 270 clinical trials [16], CTC are not really implemented in clinical practice, are generally not recognized to be of “clinical utility” [11], and many trials evaluate their impact in metastatic patients, when “it is too late”. Therefore, it is also our responsibility, as physicians and scientists working in the field of CTC, to try to understand the causes impairing the energies, time and money devoted to this topic.

## CTC Biological Characteristics and Related Challenges

The challenges that CTC tests developers face in the field of CTC are not completely disconnected from the challenges that tumor cells face in their “obstacle course” toward their metastatic “soil”, while the goals of the two “teams” are, of course, opposite.

1. Circulating tumor cells are known to be extremely rare, in the range of 1 per ml of blood, which represents, on average, one tumor cell mixed with 10 million leukocytes and 5 billion erythrocytes. This is a challenge for tumor cells as it has been demonstrated in animals that the number of circulating tumor cells correlates with the probability of metastases formation [14, 15, 17]. For CTC tests developers, this rarity and the fact that the test sensitivity is directly linked to its potential clinical benefit at early stages of the disease, is a headache. CTC are not detectable by the current blood analyses, which only screen 20 to 50 μl of blood, a volume under the CTC range. Blood is a very heterogenous liquid prone to clogging and coagulation, therefore the volume of blood that the test allows to treat is also an important parameter for the test sensitivity. One cell mixed with billions of other cells is a level or rarity which competes with the popular needle in a haystack and seems impossible to be faithfully depicted. Furthermore, if the test's sensitivity is not strictly bound to the test's diagnostic specificity, its potential clinical utility for patients is lost. Consider for example a very sensitive test to detect tumor cells in the urine in order to follow patients with bladder cancer. If the test is not also provided with a diagnostic specificity concerning tumor cells detection its chances to be of clinical utility are close to zero (Fig. 1).

**Fig. 1:**
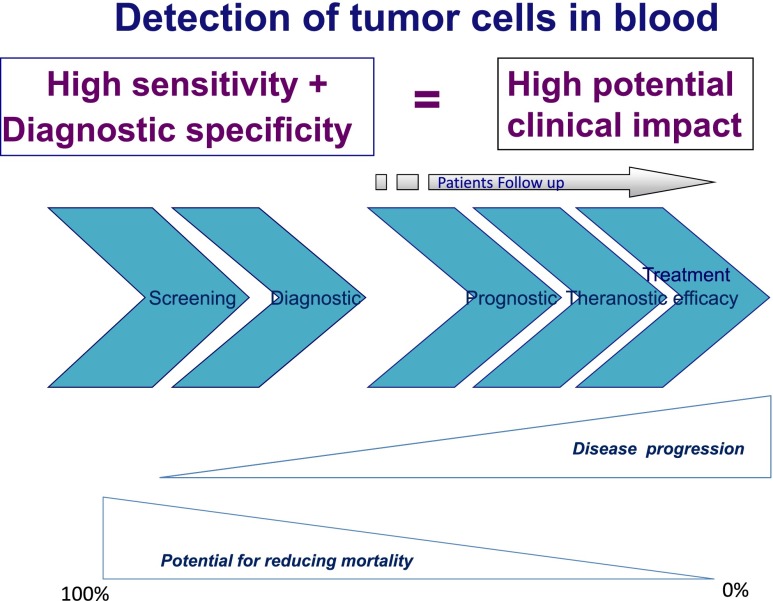
Expected clinical utility of the field of Circulating Tumor Cells in subjects at risk of developing solid cancer (*blue thick arrows on the left*) and in patients with already diagnosed solid cancer (*blue thick arrows on the right*)
2. Circulating tumor cells are fragile. Tumor cells in blood are susceptible to anoikis and exposed to attacks of the immune system and to damage by rheological conditions [13]. Additionally, they may be undergoing transformation from an epithelial to a mesenchymal phenotype (EMT) [7, 16], which is presumably linked to fragility related to massive structural changes. It is known that a relevant number of CTC die in blood before landing at distant organs [13, 15]. This is a challenge for tumor cells, and one of the main causes of their metastatic inefficiency, but is also a challenge for test developers as those precious cells can easily vanish during the attempts to extract them from blood. Moreover, their morphology can be damaged during extraction from blood preventing further cytopathologic analysis.
3. Circulating tumor cells are very heterogeneous. CTC have different levels of malignancy and capacity to found metastases. On the clinical side, this means that if we would like to use tumor cells in blood to assess more precisely the patient’s risk of developing metastases, we would need to quantify, in addition to the number of tumor cells collected from blood very sensitively without bias and counted diagnostically, the number of those cancer cells in transition from epithelial to mesenchymal phenotype (EMT) [16], those with full mesenchymal phenotype, those with “stem” characteristics, and the number of circulating tumor microemboli [8, 16]. Epithelial-to-mesenchymal transition (EMT) of carcinoma cells results in reduced expression of epithelial markers and increasing expression of mesenchymal markers, such as vimentin, also expressed in leukocytes, and N-cadherin. EMT and CTC heterogeneity represent a strong limitation for methods aiming at isolating CTC using antibodies since specific markers of tumor cells, i.e. markers expressed by all the tumor cells and not expressed at all by other blood cells or circulating non-tumor cells, are unknown at present [16].
  Besides N-cadherin and vimentin, markers of EMT include nuclear localization of β-catenin, and increased expression of transcription factors such as SNAIL, SLUG, TWIST, ZEB1, ZEB2 and/or TCF3 inhibiting the production of E-cadherin. EMT is associated with an increased cell capacity for migration and invasion, as well as resistance to anoikis and apoptosis [18, 19]. Different subsets of circulating tumor cells may have a range of intermediate phenotypes, between epithelial and mesenchymal, which could be a sign of high plasticity and “stem” character [20, 21].^.^ This plasticity is thought to be linked to genetic and epigenetic changes of cancer cells [22].
  Cancer cells with a stem phenotype (CD44^+^CD24^−/low^, ALDH1^+^) may also circulate in blood [21, 23]. Some researchers have recently been able to obtain ex vivo culture of CTC from a minority of breast cancer patients [24]. The possibility to consistently culture CTC from cancer patients would provide an extraordinary tool to test in vitro their drug sensitivity as currently performed with leukemic cells. However, the majority of CTC are expected to be out of cycle [13], not susceptible to be cultured or very resistant to re-enter the cycle. Therefore, the clinical utility of this approach is linked to the development of an assay which would 1) consistently stimulate CTC proliferation in vitro; 2) assess if the proliferating cells are really tumor cells (as circulating non-tumor cells precursors are highly susceptible to proliferate); 3) assess whether the method which stimulates CTC proliferation artificially affects, or not, CTC responsiveness to drugs.
  Some authors have demonstrated the existence of “tissue-competent” CTC for brain metastases (EPCAM^−^ expressing HER2, EGFR, NOTCH1 and HPSE), i.e. « soil-specific » seeds, opening the way to the exciting perspective of future tests predicting the type of upcoming metastasis [25]. Results obtained in animals [26] have shown that “stem tumor cells” are highly concentrated in the blood compartment as compared to the primary tumor site. If studies carried out in patients could confirm that blood contains the most malignant tumor cells genotypes, the clinical interest of using the blood tumor cells compartment for theranostic analyses would be huge. In fact, the study of different samples from primary tumor tissues has shown extensive intra-tumor genetic heterogeneity [27, 28], which could lead to biased theranostic tests performed on localized tissue sampling missing the most malignant genotypes. Cancer stem cells have also been described to undergo bidirectional transformation to a differentiated phenotype and vice versa [29], a character that could be shared by circulating cancer stem cells and make even more difficult assessing their metastatic potential.
  Another expression of CTC heterogeneity and malignancy is the possible presence in blood of circulating tumor microemboli (CTM). Originally described in mice studies [30], then in humans [8, 31–33], CTM are known to have an increased metastatic capacity. They may include stromal cells and bring “their own soil” [34]. Recently, it has been shown in animal studies that they arise from oligoclonal tumor cells groupings and not from intravascular aggregation events [35]. Finally, recent studies using a mouse model of pancreatic cancer and single-cell RNA sequencing have identified the expression of extracellular matrix genes, including SPARC, in CTC, an interesting finding that the Authors confirmed in CTC from patients with pancreatic cancer [36]. Such data demonstrate that CTC may be characterized by a very high level of plasticity, which is presumably related to their capacity of creating distant metastases. These and further studies focused on heterogeneity of tumor cells in blood should be able to shed more light on the markers associated with their increased metastatic potential.
  From a technical point of view, CTC heterogeneity represents a challenge for CTC tests developers. In fact, the optimal CTC test would require the isolation of all types of CTC without any loss allowing to further perform their immune-molecular characterization. CTC heterogeneity also highlights the difficulty of using antibodies or cocktails of antibodies to isolate/identify all types of CTC as we lack specific markers expressed in all types of CTC and not expressed in non-tumor cells [16]. There is a need for a broad-spectrum specific cocktail of cell surface epithelial and mesenchymal markers [19] covering all potential CTC phenotypes. This cocktail, however, could increase the chance that at least some of these markers cross react with, or are expressed by, blood cells and/or other circulating non-tumor cells, which would lead to false-positive results [16]. Concerning tumor-specific markers, melanoma-associated antigens (MAGE) are specifically expressed in melanoma cells. Still, they are generally used as transcripts in RT-PCR methods to detect CTC, which do not allow counting of the CTC number nor CTC immunomolecular characterization [37, 38]. Tissue-specific markers, such as prostate-specific antigen (PSA) for prostate cancer and mammaglobin for breast cancer, are not tumor-specific markers as they can be expressed in non-tumor circulating epithelial cells [39] and be down regulated during dedifferentiation of tumor cells [16]. Some markers, like HER2 and EGFR are expressed at higher levels in cancer cells as compared with normal cells in certain tumor types [9]. Since they are not expressed in all the tumor cells, these markers can be used to characterize CTC and to guide the use of targeted therapies, but are not useful for the systematic diagnosis of CTC. In fact, in metastatic breast cancers, HER2 positivity rates of CTC vary widely, between 27 and 63 %, depending on CTC isolation and characterization methods. Furthermore, up to 49 % of patients with HER2 negative primary tumors have HER2 positive CTCs and conversely, up to 77 % of patients with HER2 positive primary tumors have HER2 negative CTCs [9].
  To summarize, CTC heterogeneity is a biological characteristic of CTC and a challenge for CTC tests developers, who have difficulties finding CTC-specific markers. For CTC, their heterogeneity could limit their metastatic potential in the blood microenvironment, for instance by “diluting” the effect of platelets [40, 41] which are know to act as protection against immune-mediated lysis [42] and as EMT inducers [43].
4. Circulating Tumor Cells are not the Only Rare Cells in Blood, nor the Only Cells Derived from Organs. Early studies of CTC using prostate specific antigen (PSA)-specific [39] and alpha-fetoprotein (AFP)-specific [44] transcripts as markers in RT-PCR based assays have shown that non-tumor epithelial cells circulate in blood, for instance in patients with prostatitis [39] and in patients undergoing surgical procedures [44]. These studies also demonstrated that even the AFP transcript, which is considered a tumor-specific marker, is expressed in liver derived non-tumor cells [44], raising the issue of the best approach to identify, without mistake, tumor cells in blood. These observations prompted us to develop a method allowing the unbiased identification of CTC by cytopathological analysis [32]. Circulating epithelial non-tumor cells have been detected in blood of patients with benign colon diseases [45] and in patients with cancer [46]. They are shed in blood during inflammation, infections and invasive procedures like surgery and biopsies. Their presence in blood is a source of false positive confounding results when the CTC test targets the isolation and identification of tumor cells using epithelial markers. Non-tumor epithelial cells are not the only rare non-tumor cells circulating in blood. Circulating Endothelial Cells (CEC) and Circulating Endothelial Precursors (CEP) may be present in blood, in particular in cancer patients [47, 48], as well as stem cells of different types and lineages [49–51]. These circulating non-tumor cells represent a challenge for the differential diagnostic identification of circulating cancer cells and circulating cancer stem cells. Furthermore, functional studies targeting circulating cancer cells proliferation need to rule out the selective growth of circulating non-tumor cells.
  Overall, these findings demonstrate that the ‘perfect’ CTC marker expressed on all CTC but not expressed on blood cells, nor on other rare circulating non-tumor cells (epithelial cells, mesenchymal cells, endothelial cells, haematopoietic stem cells and mesenchymal stem cells), and never down regulated during tumor cells circulation and invasion, does not exist. In such setting, it is difficult to understand why cytopathology, which is the reference method in clinical oncology for the diagnostic identification of tumor cells, is not extensively used for the reliable detection of cancer cells in blood.

## Principal Methods for CTC Enrichment/Detection

### Methods Based on Antibodies

**Fig. 2:**
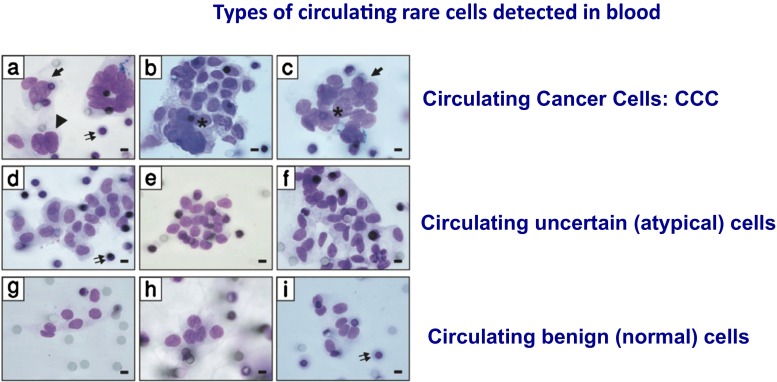
Types of circulating rare cells complicating the diagnosis of presence of tumor cells in blood. Circulating Cancer Cells (CCC) may contain tumor cells in Epithelial to Mesenchymal Transition (EMT) not expressing epithelial antigens. Circulating atypical and normal cells from organs express epithelial antigens but are not tumor cells

Marker-based technologies rely on proteins, detected by antibodies, or transcripts, detected by RT-PCR (reverse transcriptase-polymerase chain reaction). Despite the fact that an ideal “marker” or “cocktail of markers” specific to CTC is unknown at present, the majority of enrichment and detection methods are based on CTC non-specific “markers”. Epithelial markers are expressed on normal epithelia and carcinomas (epithelial tumors) but absent on mesenchymal cells including leukocytes and have been extensively used to distinguish epithelial circulating cells from normal blood cells [52, 53]. However, epithelial circulating cells include epithelial tumor cells, epithelial non-tumor “atypical” cells and epithelial non-tumor normal cells (Fig. 2). Epithelial cell adhesion molecule (EpCAM) is the cell membrane marker most frequently used for positive enrichment of epithelial cells from blood, combined with cytokeratins (CK8, CK18 and CK19), which are also epithelial cells specific markers. Enrichment of circulating epithelial cells by immunomagnetic capture is currently the most widely used approach. The semi-automated CellSearch® platform (Janssen Diagnostics, Raritan, NJ, USA) enriches epithelial cells using ferromagnetic beads coated with epithelial cell adhesion molecule (EpCAM) and has introduced a CellSearch-related definition of CTC: intact cell with a round to oval morphology and at least 4 μm in size, positive for DAPI with nucleus inside the cytoplasm (>50 %) and a nuclear area smaller than the cytoplasm, positive expression of cytokeratins and absence of the leukocyte marker CD45 [52, 53].

CellSearch® has obtained FDA approval as prognostic biomarker for metastatic breast, colon and prostate cancer [54–56]; however, despite its use as “reference test”, it shows fundamental limitations concerning sensitivity, selection bias, false positive [45] (epithelial non-tumor cells detected as tumor cells in patients without cancer and in patients with cancer) and false negative [46, 57–59] (not detected circulating mesenchymal tumor cells) results. In fact, its clinical validity has been debated. The American Society of Clinical Oncology Tumor Marker Guidelines stated that measurement of CTC by CellSearch should not be used for diagnosis or treatment modification in patients with breast cancer [60]. The large majority of studies showing the prognostic value of CellSearch are performed in patients with metastatic cancer, which is not the most appropriate clinical setting to assess the benefit of the CTC field on patients’ mortality. A large-scale pooled analysis of thousands of patients with breast cancer [61] showed a prognostic value of CTC both in M0 and M1 patients. However, in this study, the majority of M0 patients were analyzed by RT-PCR, and only one out of the 18 studies performed with CellSearch was performed in M0 patients. Raimondi et al. [11] have recently provided an extensive review of the clinical trials carried out and ongoing with CellSearch after the SWOGS0500 trial has failed to demonstrate the clinical utility of counting CTC by CellSearch to assess the effectiveness of frontline chemotherapy in metastatic breast cancer patients. They conclude that the clinical utility of the CellSearch « concept » is still suspended in « Limbo », which is not surprising given the test’s technical limitations. Chinen et al. [62] have published the important observation that the selective detection of epithelial cells can skew our assessment of patients response to treatment. By assessing the CTC number in parallel with an epithelial-mediated cell capture and with ISET®, which provides unbiased isolation and diagnostic detection of tumor cells, during a lung cancer patient’s follow up, they have shown the disappearance of epithelial cells upon therapy. However, simultaneously, ISET® found an increasing number of CTC, detected by cytopathology, since mesenchymal tumor cells, resistant to treatment, underwent expansion leading to the patient’s death.. Clearly, assessing CTC with an incomplete and non-diagnostic test can introduce a bias in the assessment of therapeutic efficacy.

EpCAM antibodies can be used to attach epithelial cells to columns and microposts, which adds a cell selection based on cell deformability, or to magnetic devices. These alternative EpCAM-based enrichment technologies include microfluidic devices CTC-chip [63], Herringbone chip [64], iCHIP [65] and IsoFlux [66] and MagSweeper [67]. GILUPI [68] is a medical wire internally coated with anti-EpCAM antibodies, which is placed directly into the antecubital vein for 30 min in order to sample a large blood volume.

The technologies based on EpCAM-mediated capture demonstrate limitations which are similar to those of CellSearch, i.e. false positive and false negative results and lack of diagnostic assessment of CTC, which are problematic in the clinical setting. However, some of them provide living epithelial cells, for further analyses and in vitro growth.

### Methods Based on Transcripts

RT-PCR-based detection of specific transcripts is quite different from capture and visualization of intact cells, and low-level illegitimate expression of the targeted transcript can lead to false-positive results [13, 69]. RT-PCR based detection methods are performed on the total RNA extracted from blood with or without previous separation of leucocytes or after cell capture. For instance Adnatest [70] “breast” captures cells with Epcam and MUC1 antibodies then performs transcript RT-PCR based analysis. These tests however do not allow cell counting and are not diagnostic. In a study using RT-PCR specific to Alpha-fetoprotein (AFP) transcripts we have demonstrated that even a well know marker considered to be a “tumor-specific marker” (AFP) is expressed in non-tumor circulating liver-derived cells [44].

### Methods Based on Functional Tests

Two different in vitro assays have been optimized to detect viable cells obtained from cancer patients: the EPISPOT assay, which implies selection with Rosettesep epithelial specific antibodies and detects cytokeratins 19 [71] secreted during in vitro culture, and an invasion assay assessing the ability of cells to digest a fluorescently labelled cell adhesion matrix [72]. Cells are first isolated using other methods before assays are applied to examine their function. These methods target viable cells characterization but are not diagnostic for tumor cells, due to their use of non-tumor specific markers or functions like phagocytosis, and their sensitivity is decreased by the need of blood pre-treatment.

More complex functional studies have been performed by transplantation of circulating cancer cells into immunodeficient mice [21]. The EpCAM negative metastases that were grown (EPCAM^low^MET^high^CD47^high^CD44^high^ phenotype) were derived from EpCAM negative tumor cells, demonstrating that, in this study, metastasis-initiator cells are EpCAM negative [21] and not detectable by EpCAM based tests. These data are consistent with the increased malignant potential of tumor cells loosing epithelial antigens during EMT and/or because of their “stem” character and with the general view that EpCAM postive cells, currently selectively detected by the majority of CTC-tests, are not expected to be the metastasis-initiator cells. Another study, however, showed that CTC from patients with chemosensitive or chemorefractory small-cell lung cancer were tumorigenic in immunocompromised mice, and that the CTC-derived explants mirrored the response of the donor patient to the treatment [73]. In this study, tumour cells were enriched from blood using the RosetteSep™ enrichment cocktail which is designed to enrich tumor cells by negative selection. Therefore, epithelial and non epithelial tumor cells may have been injected into mice. The Authors underline indirect evidence that tumorigenic cells could be epithelial cells, based on genetic analyses, correlation of the number of epithelial cells detected by CellSearch and CTC-derived explants growth rate and previously established prognostic value of circulating epithelial cells detected by CellSearch. However, an assessment of epithelial and non epithelial circulating tumor cells in the patients blood used for the study was not performed leaving the question of the type of metastasis-initiator cells unanswered. Although conceptually very interesting, these in vivo assays require very high numbers of CTC, which limit their applicability in clinical settings in the absence of efficient and reproducible methods to culture CTC.

### Methods Based on Negative Selection

Since techniques based on positive-selection using markers share the common drawback of capturing only a fraction of the heterogeneous CTC population, and face the unresolved challenge of the lack of tumor-specific markers, methods based on negative selection, in which the blood sample is depleted of leukocytes, have been developed [65] to avoid loss of the most malignant CTC with high phenotypic plasticity. Using magnetic beads with antibodies against CD45 and/or CD15 that bind to leukocytes, these cells are then removed by placing the sample in a magnetic field [19, 74, 75]. Some Authors use bi-specific antibodies against antigens on leukocytes and erythrocytes that induce the formation of large multicellular rosettes, which can be easily removed from blood by Ficoll density centrifugation [71]. However, cells that are positive for both cytokeratins and CD45 have been detected in the blood of patients with carcinoma. Their significance is unknown; they could be artifacts related to the sample’s pre-analytic processing [53, 76] or macrophages after phagocytosis of epithelial cells [77]. Furthermore, not all CD45^–^ cells in blood are tumor cells. Circulating endothelial cells [78], non-tumor stem cells and non-tumor epithelial cells are CD45^–^. Subsequent diagnostic approaches are needed to increase assay performance and specificity. One concern for this approach is sensitivity as, due to the huge difference in number between leucocytes and CTC, leukocytes capture can drag some unlabelled tumor cells. Finally, the purity of the recovered samples is low and it is difficult to identify CTC amongst millions of leukocytes.

### Methods Based on Physical Characteristics: Cell Size

#### ISET®

**Fig. 3:**
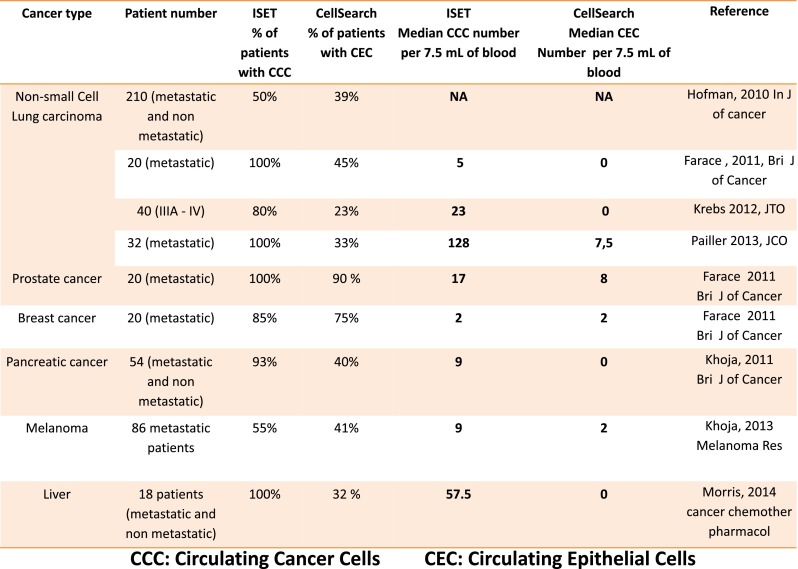
Summary of comparative studies targeting CTC detection by using ISET and CellSearch

ISET® (Rarecells Paris, France) is the first method based on blood filtration [32]. Initial studies of CTC using PSA-specific [39] and alpha-fetoprotein-specific (AFP) [44] transcripts as markers in RT-PCR based assays had shown that non-tumor epithelial cells circulate in blood, in patients with prostatitis [39] and in patients undergoing surgical procedures [44]. These studies also demonstrated that even the AFP transcript, which was considered a tumor-specific marker, is expressed in liver derived non-tumor cells [44], and that EpCAM is expressed only in a proportion of tumor cells in tumor tissues [79]. These data prompted the development of an approach able to isolate tumor cells from blood avoiding the use of cellular markers. The ISET® (Isolation by Size of Epithelial Tumor cells) method [32] is based on the principle that, according to cytopathological criteria [80], tumor cells from organs are much larger than leucocytes, with nuclei of 24 μm or larger [80, 81]. They are therefore retained on the ISET® filter, while the majority of leucocytes are lost through the 8 μm pores. The acronym ISET® was referred to Isolation by Size of Epithelial Tumor/Trophoblastic cells when it became clear that the method can extract from maternal blood the very rare circulating trophoblastic cells [82–85]. It became referred to Isolation by SizE of Tumor/Trophoblastic cells recently since several scientific teams demonstrated that it also sensitively isolates non-epithelial tumor cells, including tumor cells in EMT [33, 57, 58, 86–89], from uveal melanoma [90, 91], from cutaneous melanoma [92–94] and from sarcoma [95]. The characteristic of ISET® is the very sensitive isolation from blood of circulating rare cells, keeping them intact, thus allowing their cytopathological analysis. Blood filtration with these aims is not trivial: ISET® is based on 30 parameters, located in the buffer, filter, cartridge and device, which are tuned to obtain the ISET® technical specifications. Using ISET® and cytopathology, the team of Dr. Hofman has shown the possibility to distinguish, on ISET® filters, cells with malignant phenotype (CTC) from cells having uncertain malignant phenotype and cells having benign phenotype (Fig. 2) [46, 80, 81]. These studies not only demonstrated that the cytopathological analysis of cells extracted from blood by ISET® is possible, but also that the blood of cancer patients may contain a mixing of cells from organs which are not all tumor cells and are thus expected to introduce a bias in results of CTC tests using epithelial markers. Dr Hofman’s team then considered the question if the classical cytopathological criteria used in exfoliative cytology, fine needle biopsy, Pap test and other cytopathological tests are still valid when applied to cells isolated from blood using ISET®. This question was relevant as, before ISET®, only haematological, not cytopathological, analyses were performed on blood. Dr Hofman’s team coordinated a study on ISET® filters obtained from 770 subjects, including 569 patients with cancer and 201 subjects without cancer, including healthy donors and patients with benign pathologies [80]. Ten pathologists examined blindly and in parallel the 770 filters, without knowledge of the patients/subjects clinical data. They accurately identified tumor cells in patients with cancer and not in patients without cancer. However, results were consistently wrong for seven patients with thyroid adenoma and three patients with parathyroid adenoma. Since these specific pathologies are known as not being suitable for cytopathological analysis and this aspect is universally known, it was concluded that the cytopathological analysis of cells extracted from blood by ISET® can rely on the same criteria used by classical cytopathology and that cytopathology on cells from organs isolated from blood by ISET® is expected to have the same diagnostic reliability  and the same limitations than classical cytopathology. These results clearly showed that patients can benefit from a cytopathological diagnosis of circulating tumor cells. It has to be highlighted that tumor cells isolated by ISET® and identified by cytopathology are only in part the same cells identified by CellSearch [33, 46, 57–59, 86–89], as the cytopathological reading includes tumor cells in EMT isolated by ISET® (but not isolated by CellSearch), and excludes epithelial non-tumor cells (which are counted by CellSearch). Furthermore, ISET® has been shown to be more sensitive than CellSearch [57, 59, 86, 88, 89, 94] (Fig. 3). As a consequence, we will refer to these cells isolated by ISET and identified by cytopathology as Circulating Cancer Cells (CCC) in order to distinguish them from CTC. CCC are cancer cells extracted from blood potentially without loss and/or bias and diagnostically identified by cytopathology.

It is very difficult to assess the sensitivity of different methods to isolate CTC based on published non-comparative data as they rely on variable non-standardized models and protocols. As an example, Vona et al. published in 2000 the results of a very accurate spiking test showing that the sensitivity threshold of ISET® is one tumor cells per ml of blood [32]. This threshold level has been subsequently confirmed by several ISET® users [86, 90, 92]. In 2004, Allard et al. published the sensitivity of CellSearch which was reported to be similar and a little higher: 4/4 cells found in 7,5 ml of blood [52]. As shown in Fig. 3, subsequent comparatives studies performed in vivo have established that the sensitivity of ISET® is consistently higher than the sensitivity obtained using CellSearch [57, 59, 86, 88, 89, 94]. Furthermore, it is the combination of sensitivity and diagnostic specificity which is of clinical value. The current sensitivity threshold of ISET® is 1 cell in 10 ml of blood (Laget S et al., manuscript in preparation) for isolation of fixed and unfixed cells.

Some Authors [16] question that ISET® may loose “smaller” (than 8 μm) tumor cells on the basis that the definition of CTC by CellSearch is a cell of 4 μm or larger [52, 53]. We think that non diagnostic criteria for tumor cells [52, 53] should not prevail on cytopathological diagnostic criteria which have been clinically validated for the diagnosis of cancer cells over the last century. Breast cancer cells, for instance, which are considered small tumor cells, have a mean diameter ranging from 29.8 to 33.9 μm [96]. Tumor cells from “Small Cell Lung Carcinoma” have been reported to be twice in size as compared to mature lymphocytes [97] (thus 16 μm). Others hypothesize that ISET® may lose circulating tumor stem cells (personal communications). This question has to be answered. However, cells from stem cell lines are on average 12 μm in size (P. Paterlini Bréchot unpublished), and are sensitively captured by the 8 μm ISET® pores. Consistently, trophoblastic cells, which size is 12–15 μm, are very sensitively captured by ISET® [82–85],

#### Other Methods Based on Physical Characteristics

Several different methods have been reported recently which avoid the use of makers at the isolation step and are currently under evaluation [8, 16]. Major challenges for these new developments are cell fragility, especially for chips isolating living cells, blood tendency to clogging, high blood cellularity and use of physical properties which are not completely specific for tumor cells like deformability. Relevant parameters are sensitivity, purity (depending on the rate of leucocytes contamination), blood volume which can be analysed, time for isolation, quality of cell morphology and cell structure for further morpho-immuno-molecular analyses, and viability for further culture tests.

A microfluidic spiral biochip (Parsortix) [98] has been developed in which blood samples are made to flow through microscopic channels and cells are separated into sub-populations based on their flow rate. CTC having larger size and less deformable structure as compared to normal blood cells are expected to move through these channels at a different rate from leucocytes and are extracted from collection areas. However, such intricate microfluidic devices are typically affected by blood clots and air bubbles. Furthermore, CTC capable of undergoing EMT might also be as deformable as leukocytes [16].

A new technology currently undergoing clinical validation is the use of dielectrophoresis (DEP) to isolate CTC [99–101]. Cells including CTC are typically electrical insulators, but they can be polarised when a specific non-uniform electrical field is applied on them in a liquid, so they act as ‘dielectric particles’. In a DEP device, cells in a sample are polarised and then subtle changes in the strength of electric fields can be made to make the cells move to a collection center. As CTC have unique membrane properties and size amongst blood cells, they can be specifically polarised and moved.

## Molecular Characterization of Circulating Tumor Cells

**Fig. 4:**
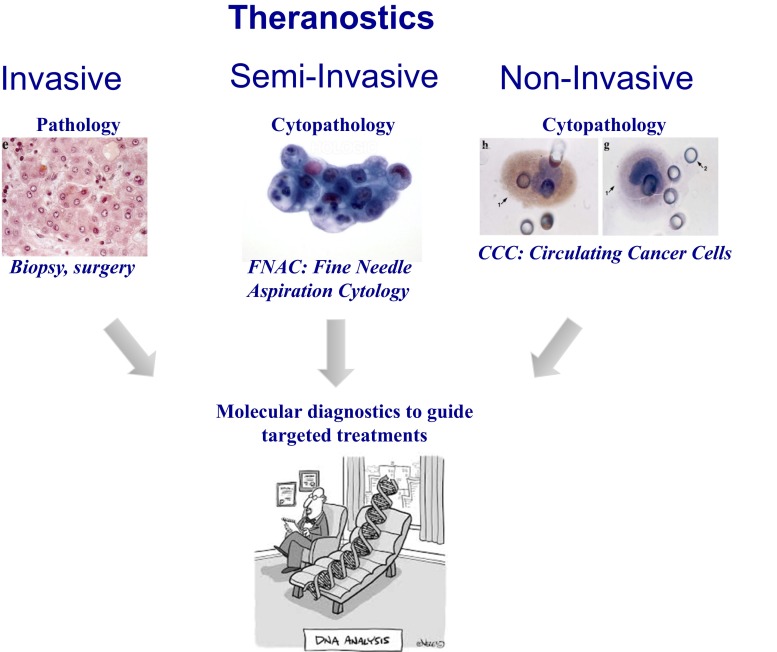
Invasive, semi-invasive and non-invasive theranostic analyses rely on pathological and cytopathological identification of tumor cells

Molecular analyses of CTC are not in the scope of this article. However, this is a hot and important field to understand the mechanisms of tumor invasion and explore the impact of genetic analyses for clinical theranostic use [8, 16, 88, 93, 102]. Most studies use whole-genome amplifications for single cell studies targeting the CTC heterogeneity. Other studies pool CTC from the same patient sample to study the whole tumor cell population. A key issue for these analyses, from a clinical point of view, is the tumor nature of the cells which are the target of molecular analyses. It seems clear that targeting genetic studies to cells which are not diagnosed as tumor cells is bound to the risk of obtaining biased results. For instance if single cells analyses, or pooled cells analyses, are targeted to cells identified as “tumor cells” only on the basis of their epithelial antigens, the approach cannot ensure that results are reliable. This issue is of course highly relevant if targeted treatments are selected or not based on non-invasive genetic analyses of circulating tumor cells. We believe that cytopathological identification of tumor cells extracted from blood should be performed in order to target molecular analyses to diagnostically validated Circulating Cancer Cells and follow the same principles of invasive and semi-invasive, (through Fine Needle Aspiration Cytology (FNAC)) theranostics (Fig. 4).

## Key Questions

### Can we Diagnostically Identify a Tumor Cell Using a Different Approach than Cytopathological Analysis?

**Fig. 5:**
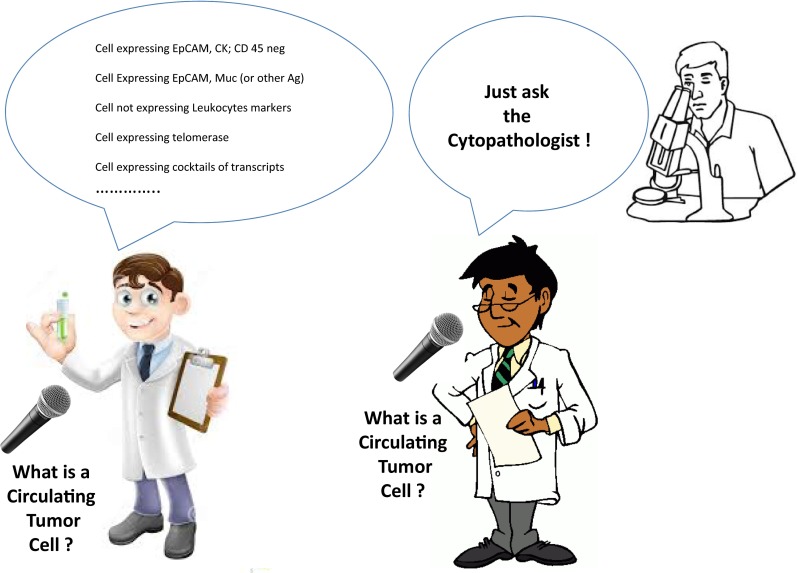
Different possible perceptions of “what is a Circulating Tumor Cell” according to “CTC test developers” and “CTC test users”

As previously mentioned, “ideal” specific markers of tumor cells do not exist [16]. Some markers, like HER2 and EGFR are expressed at higher levels in cancer cells as compared with normal cells in certain tumor types [9], but it remains questionable if a different level of expression is, by itself, diagnostic of the tumor cell nature. Typically, it is considered that the presence of mutations is a reliable marker of the tumor nature. However, recent studies have clearly demonstrated the presence of oncogene mutations in benign pathologies [103, 104]. Tumorigenesis is known to occur through accumulation of genetic mutations [105]. The view that “presence of mutation” is, in itself, a “demonstration of the tumor cell nature” has to be critically assessed. Therefore, up to the moment when other cell diagnostic criteria will be extensively clinically validated, only cytopathology is expected to ensure a reliable diagnosis of tumor cells, including tumor cells derived from solid tumors and present in blood (Fig. 5). Continuous efforts combining cytopathology and molecular analyses are expected to bring developments to this field.

### Can the Study of CCC decrease mortality Cancer Patients?

The study of Circulating Cancer Cells, i.e. tumor cells which have been isolated from blood without loss and/or bias and which have been identified diagnostically by cytopathological analysis, brings the hope of reducing cancer mortality through early diagnosis of tumor invasion, early detection of treatment efficacy/failure and, possibly, early diagnosis of invasive cancers. Clinical trials have now to be implemented to assess the clinical potential and benefit of CCC detection, counting and characterization. In parallel, many aspects of the CCC field should be further investigated to expand our understanding of tumor invasion. These aspects include the circadian rhythms of CCC circulation, the role of physical activity and/or changes in the blood flow rate (as in postprandial times) on CCC circulation. We also do not know if and how CCC spreading may vary according to the tumor growth rate, the tumor angiogenesis, or both factors combined. Seemingly, it is unknown if CCC spreading proceeds by “waves” or occurs continuously and which is the impact on CCC circulation of disease-related (stage, histological type, etc..) and patient-related (general conditions, immune response efficiency/impairment, etc..) factors.. Finally, we do not know which is the life span of CCC in blood and if it is related to the tumor tissue type/subtype and/or to their phenotypic and genotypic characteristics. These relevant issues should be investigated by future studies to help the clinical application of the CCC field.

### Can CCC Allow Early Diagnosis of Solid Tumors?

In 1995 Kohn and Liotta [106] published data showing that in situ breast cancer is a clonal precursor of breast carcinoma and that tumor invasion starts 5 to 10 years before cancer diagnosis. This raised the possibility, and hope, to detect cancer at a pre-diagnostic step through the very sensitive and diagnostic detection of “sentinel” cancer cells in blood. It is known that early diagnosis of cancer gives the best chance to obtain its eradication, as demonstrated by Pap-test and early diagnosis of cervical cancer; therefore, this route represents a clear hope to reduce cancer-related mortality. In 1992 Rhim et al. [26] published results obtained in a mouse model of pancreatic cancer showing that tumor cells can circulate in blood when the primary tumor is still undetectable. However, detection of cancer cells in blood at a so early stage was never demonstrated in humans before the recent study of Ilie M et al. [107]. These Authors followed a cohort of patients with COPD (Chronic Obstructive Pulmonary Disease), a pathology carrying an increased risk to progress to lung cancer, with yearly CT-scan and ISET® analysis searching for CCC. Five out of 168 COPD patients showed CCC while the CT-scan performed in parallel did not show any nodule. A lung nodule was found by CT-scan 1 to 4 years after the detection of CCC by ISET® and the five patients underwent prompt surgical resection of the nodule. The pathological analysis of the removed nodule confirmed the presence of a lung cancer: all the patients were diagnosed at stage 1A. The 16 months follow up of these five patients did not show any sign of cancer - nodule, lymph nodes, CCC- allowing to hope that lung cancer, detected and treated at a very early stage, had been eradicated. Clearly larger multicenter studies are now needed to expand this study. However, both the specificity and sensitivity of the study were 100 % as no patient without CCC nor patient in the control cohort developed cancer. This study is therefore a proof of principle that a very sensitive and diagnostic approach to detect tumor cells in blood (CCC identified by cytopathological analysis) could reduce mortality through the very early diagnosis of invasive cancers. This study also demonstrates that the ISET® test’s characteristics of sensitivity and diagnostic specificity are closely linked to its potential clinical utility.

It is thought that tumors which are detectable by current imaging analyses usually contain more than 10^9^ tumor cells [16]. If the tumor is invasive enough to spread cancer cells, we can take advantage from them as the tumor’s “Achilles heel” to set up a close surveillance and remove the tumor as soon as it becomes detectable by imaging. An important issue concerning lung cancer screening is that CT-scan has been shown to detect lung nodules with 96 % false positive results [108]. Since biopsy of lung is not devoid of morbidity, doctors often prefer to wait and repeat the CT-scan after months looking for changes in the nodule shape and/or size further suggesting the presence of lung cancer. However, this strategy allows lung cancer to progress. In this setting, it would be important for patients with a nodule in the lung to benefit from a sensitive blood test, able to reliably detect CCC, as the presence of cancer cells in blood, combined with the detection of a nodule by CT-scan, increases the probability that the nodule is a small cancer and allowing its early surgical resection.

The “natural history” of early CCC spread in the blood of patients with different types of cancer is not known and is awaiting further studies. It should be investigated whether tumors consistently start diffusing CCC at early steps of their development and how this process proceeds over time during tumor growth. These studies could explain why not all the patients diagnosed with cancer have detectable CCC. Furthermore, the genotypic and phenotypic characteristics of these CCC detected in blood at a very early stage are unknown. Could we envisage early targeted treatments or treatments specific to cancer stem cells at a very early stage of cancer development? Our limited knowledge of this field limits our understanding and the possible clinical use of CCC for early diagnosis of solid cancers. However, these preliminary results should foster more fundamental investigations and clinical trials.

## Shedding Some Light on the Fog

### Circulating Tumor Cells are Cells, but they are generally referred to as « Biomarkers »

It is difficult to understand why « CTC » are considered a « biomarker » while they are cells. A biomarker is generally a molecule, like PSA or AFP, and defined as “any substance, structure, or process that can be measured in the body or its products and influence or predict the incidence or outcome of disease” [109]. Cells are generally not considered biomarkers and cytology is rather compared with biomarkers [110]. “Biomarkers” have cut off, which is not the case of cytopathology, which is diagnostic. It is noteworthy that, considering the biological characteristics of tumor cells in blood, including their heterogeneity and complexity, a cut off for their clinical significance is surprising. In particular, a cut off related to epithelial (EpCAM positive) cells in blood, which do not include the most malignant tumor cells in EMT and which may include epithelial non-tumor cells, for instance after surgical interventions or during inflammation, seems to be a very imprecise and limited parameter.

### The Term Circulating Tumor Cells for Tests Based on Epithelial Markers Introduces a Terminological Bias

In 2002, Fehm and al. published a paper [111] using the Immunicon’s Epcam based test for Circulating Epithelial Cells (CEC), which further became the CellSearch test, with title: « Cytogenetic evidence that circulating epithelial cells in patients with carcinoma are malignant ». The Authors found genetic abnormalities in CEC isolated from some patients and claimed that this is the proof of the tumor nature of CEC. However, these findings do not demonstrate that all CEC are malignant. In fact, further studies demonstrated that “CTC” detected by CellSearch may be non-tumor CEC, i.e. false CTC [45]. If we would apply the same principle to mesenchymal cells in blood, we would state that genetic abnormalities have been identified in circulating mesenchymal cells [88]; however, this would not demonstrate that all the circulating mesenchymal cells are tumor cells. Nevertheless, starting from 2002, the CEC detected by CellSearch became « CTC ». « CTC » detected with CellSearch or other tests based on epithelial antigen detection, are CEC, including possibly circulating epithelial tumor cells, possibly circulating non-tumor atypical and normal epithelial cells and excluding circulating EMT and mesenchymal tumor cells [13]. This observation, combined with a critical view of “cut off” numbers related to CEC detected by CellSearch, could explain why the concept of clinical utility of CellSearch is presently suspended in Limbo, as pointed out by Raimondi et al., [11]. However, our strong belief is that a lack of stringency in the terminology related to the detection of tumor cells in blood is susceptible to hinder the work of scientists and clinicians, in particular concerning the assessment of the clinical utility of detection of tumor cells in blood. In other words, it creates a fog which impairs the objective interpretation of results. A particular concern is related to molecular analyses targeted to “CEC” called CTC without proof that the analyses are addressed to diagnosed tumor cells collected from blood without bias. In this setting, if the proportion of epithelial non-tumor cells is increased in blood for whatever reason (inflammation, previous surgery etc..), the result of genetic analyses targeted to CEC could be incorrect an lead to a wrong choice of treatment. As mentioned, a mixture of non-tumor epithelial cells may be present in blood [46, 80] and its variable proportion versus epithelial tumor cells may have an impact on genetic results. Furthermore, the most malignant mesenchymal tumor cells, including the circulating stem tumor cells,  are not detected by tests using epithelial markers, so that their genome is not included in the genetic profiling of circulating epithelial cells, called CTC. Therefore, a non-diagnostic and partial detection of cancer cells in blood may be source of relevant clinical bias with possible adverse effects on the health of cancer patients.

In conclusion, we have underlined technical and terminological issues related to the presence of tumor cells in blood which are susceptible to hinder the use of this field to improve cancer patients diagnosis, follow up and treatment. In order to progress, the term Circulating Cancer Cells (CCC) should be used to designate tumor cells extracted from blood without bias and identified diagnostically by cytopathological analysis. CCC are to be distinguished from CTC, as this term is applied to epithelial cells extracted from blood, including tumor but also non-tumor epithelial cells and excluding circulating non epithelial tumor cells in EMT and stem tumor cells. We think and hope that this shift could help as, up to now,  much time, energy and money have been spent in the last 15 years on studies targeting CTC and yet the “killer” cancer cells keep on running in the street and killing patients.
